# Editorial: Levothyroxine Therapy in Patients With Hypothyroidism

**DOI:** 10.3389/fendo.2021.734895

**Published:** 2021-07-20

**Authors:** Alessandro Antonelli, Leonard Wartofsky, Paolo Miccoli

**Affiliations:** ^1^ Department of Surgical, Medical and Molecular Pathology and Critical Care, University of Pisa, Pisa, Italy; ^2^ MedStar Health Research Institute, MedStar Washington Hospital Center, Washington, DC, United States

**Keywords:** levothyroxine, hypothyroidism, thyroid-stimulating hormone (TSH), tablet L-T4, soft gel capsule L-T4, liquid L-T4, malabsorption

Hypothyroidism is a condition that is more frequent in women, particularly those older than 60 years of age. It is readily diagnosed by the measurement of blood levels of thyroid-stimulating hormone (TSH) and thyroxine (T4) (1). Common causes of hypothyroidism include (2–4): A) a low iodine intake in iodine deficient areas; B) autoimmune thyroiditis (the principal cause in iodine sufficient areas); C) total thyroidectomy; D) radioiodine therapy as for therapy of hyperthyroidism; E) drugs (i.e., immune checkpoint inhibitors, and tyrosine kinase inhibitors) (5–7); F) other rare causes (8).

Levothyroxine (L-T4) is a synthetic hormone with a chemical structure similar to natural endogenous T4, that is prescribed at the dose of 1.5-1.7 μg/kg body weight/day as substitutive therapy for any of the conditions associated with hypothyroidism. The treatment will lower elevated levels of TSH into the normal range for virtually all hypothyroid patients, with normalization as well of circulating free triiodothyronine (FT3) and free T4 (FT4) levels (1, 9). Nevertheless and owing to various interfering issues, ~20-50% of patients do not have an optimal response to this therapy (10, 11), and require titration with higher dosage while monitoring TSH and thyroid hormones levels (12). Pseudomalabsorption of L-T4 caused by poor patience adherence to the prescribed dosage must be excluded, and having done so, then a decreased intestinal absorption of L-T4 associated with either intrinsic gastrointestinal diseases or pharmacological interference with absorption, remains the primary explanation for refractory hypothyroidism (11).

In the twelve papers that constitute this Research Topic, various innovative aspects related to therapy of hypothyroidism with L-T4 are reviewed and discussed and provide a stimulating overview of the present state of our knowledge.

The review by Fallahi et al. discusses novel L-T4 formulations such as the liquid preparation (that does not need the dissolution of the tablet preparation in the acid gastric environment) and the soft gel capsule (that dissolves quickly in the stomach), both of which having been developed to potentially bypass the problem of refractoriness reported with “usual formulations” of L-T4.

The use of the soft gel capsule of L-T4 has been studied in some clinical trials with promising results in subjects with gastric- or coffee-associated T4 malabsorption (13), as well as in hypothyroidism without demonstrable malabsorption.

Liquid L-T4 can avoid possible absorption interference related to coffee or food that has been reported with L-T4 tablets, and may overcome the problem of malabsorption observed in the presence of an elevated gastric pH as in atrophic gastritis (14), or with proton pump inhibitors treatment, or in subjects infected with Helicobacter pylori (HP) (15). Moreover, the liquid L-T4 preparation can bypass the malabsorption in subjects undergone to bariatric surgery (16), or with lactose intolerance (LI) (17). Ideally a liquid, rapidly absorbed preparation could maintain desirable TSH levels (in the reference range) in hypothyroid patients regardless of malabsorption, gastric disorders, or drug interference (18, 19), or in patients with intestinal parasites, gluten sensitivity, or celiac disease (CD).

The review by Virili et al. focuses on the state of our knowledge of the pathophysiologic pathways that determine the absorptive fate of the tablet versus alternative L-T4 formulations (softgel capsule and liquid solution) in patients with gastric disorders.

The review by Benvenga deals with the different medications which can interfere with L-T4, discussing also the costs associated with the frequent evaluation of TSH levels when L-T4 is administered contemporaneously with the interfering drug, and the metabolic and/or cardiovascular complications observed in undertreated hypothyroidism.

While the link between subclinical hypothyroidism and cardiovascular diseases has been suggested by many studies (20), the L-T4 treatment of subclinical hypothyroidism to decrease cardiovascular disease risk has not been proven to be necessarily advantageous. As reviewed in the paper by Sue and Leung, until now most of the international societal guidelines suggest that treatment decisions should be personalized according to the patient age, cardiovascular risk, degree of circulating TSH elevation, symptoms, and comorbidities. Moreover, caution should be taken in beginning L-T4 therapy for subclinical hypothyroidism in the elderly, as also reported in the paper by Effraimidis et al. The comorbidities that are present in older patients confound the correct diagnosis with that of non-thyroidal illness. It is controversial whether the treatment of such mild forms of hypothyroidism can ameliorate mortality, morbidity, and quality of life in elderly.

In their paper, Zijlstra et al. determine the effects of L-T4 therapy on cardiovascular outcomes in older adults with subclinical hypothyroidism. The reported findings indicated that L-T4 did not significantly change the risk of cardiovascular outcomes in older adults with subclinical hypothyroidism, regardless of a history of cardiovascular disease and age.

As described in the paper by Giuffrida et al., cystic fibrosis (CF) represents another cause of intestinal L-T4 malabsorption and the novel formulations of L-T4 may be effective in hypothyroid patients to overcome malabsorption. The data suggest that L-T4 in oral liquid formulation can overcome, at least in part, the reduced absorption of L-T4 in CF patients, leading to more tailored choices and a better management of hypothyroidism.

Patients who have undergone either lobectomy or total thyroidectomy represent another unique population with challenges to the attainment of an optimal L-T4 dose to restore euthyroidism (21, 22). The paper by Miccoli et al. discusses the multiple issues related to L-T4 treatment that apply to thyroidectomized patients. In spite of the attempts to devise a therapeutic scheme or reproduceable formula able to predict the exact dosage of L-T4 required after total thyroidectomy, it has not been possible to do so.

Of patients undergoing thyroid surgery, those with thyroid cancer (TC) represent a unique and heterogeneous population in regard to subsequent L-T4 dosage in that optimal dosage will likely depend on whether or not there is residual tumor post-operatively and hence the need for TSH-suppressive L-T4 therapy rather than replacement dosage. The study by Dou et al. intends to determine the proportion of patients among those with low-risk papillary TC (PTC) who do not need substitutive or replacement therapy based on the presence of risk factors for post-lobectomy hypothyroidism. Of note in this regard was their conclusion that patients with lower preoperative TSH levels undergoing lobectomy and without Hashimoto’s thyroiditis, are more likely to have a normal thyroidal function in the first year after thyroidectomy.

Epidemiologic issues surrounding the incidence of hypothyroidism are examined in the paper by Kim et al. which offers an evaluation of both the incidence of hypothyroidism and a determination of the factors associated with hypothyroidism that lead to the need for thyroid hormone replacement. The Authors suggest that patients with an elevated risk of postoperative hypothyroidism should be made aware of their risk factors and should be monitored more intensively.

Prior to the availability of recombinant human TSH (Thyrogen) for the preparation of TC patients for radioiodine scanning and/or therapy and as still done in many medical centers, L-T4 therapy was withdrawn for 4-6 weeks in order to elevate endogenous TSH sufficiently to stimulate isotope uptake. Such withdrawal is of course a cause for hypothyroidism, albeit of limited duration. It has been noted that TC patients often display mood disorders after the withdrawal of L-T4, but it is still not known whether the disorders are related to the withdrawal of L-T4 per se or to the duration of the withdrawal or indeed some other possible mechanism. The paper by Wu et al. investigates the abnormal regional cerebral glucose metabolism (rCMRglu) in PTC patients without L-T4 for 4 weeks, in an attempt to explain the mechanism leading to mood disorders associated with transient hypothyroidism.

Recent large population studies have suggested that the high TSH levels in the range seen in subclinical or clinical hypothyroidism are associated with an increased mortality in patients treated with L-T4, independent of patient age (23, 24). These studies stimulate the need to examine this issue in future large prospective population studies in patients treated with L-T4.

Importantly, although modern approaches to L-T4 therapy has been in use for more than 60 years, cross-sectional studies have reported that ~45% of patients are either over- or under-treated. As extensively discussed in the paper by Antonelli et al., in the long term follow-up liquid L-T4 maintains normal TSH values more efficiently than L-T4 tablets, both in patients with no malabsorption, or in those with a malabsorptive state.

In conclusion, novel oral L-T4 preparations (soft gel capsule and liquid formulation) represent the most recent and welcome advances in levothyroxine therapy, and the content herein of this Research Topic provides an extensive update of the literature extant, and suggests potential directions for future research.

## Author Contributions

AA, LW and PM have equally contributed to the draft of the Editorial. All authors contributed to the article and approved the submitted version.

## Conflict of Interest

The authors declare that the research was conducted in the absence of any commercial or financial relationships that could be construed as a potential conflict of interest.
